# Viral Proteins U41 and U70 of Human Herpesvirus 6A Are Dispensable for Telomere Integration

**DOI:** 10.3390/v10110656

**Published:** 2018-11-21

**Authors:** Darren J. Wight, Nina Wallaschek, Anirban Sanyal, Sandra K. Weller, Louis Flamand, Benedikt B. Kaufer

**Affiliations:** 1 Institut für Virologie, Freie Universität Berlin, Robert von Ostertag-Straße 7-13, 14163 Berlin, Germany; aanirbansanyal@gmail.com; 2 Institute for Molecular Infection Biology, Julius-Maximilians-Universität Wϋrzburg, Josef-Schneider-Straße 2, 97080 Wϋrzburg, Germany; nina.wallaschek@uni-wuerzburg.de; 3 Molecular Biology and Biophysics, UConn Health, 263 Farmington Avenue, Farmington, CT 06030-3205, USA; weller@uchc.edu; 4 Department of Microbiology and Immunology, CHU de Québec, Université Laval, Quebec, QC G1V 4G2, Canada; louis.flamand@crchudequebec.ulaval.ca

**Keywords:** human herpesvirus 6A, HHV-6A, telomere integration, latency, recombination

## Abstract

Human herpesvirus-6A and -6B (HHV-6A and -6B) are two closely related betaherpesviruses that infect humans. Upon primary infection they establish a life-long infection termed latency, where the virus genome is integrated into the telomeres of latently infected cells. Intriguingly, HHV-6A/B can integrate into germ cells, leading to individuals with inherited chromosomally-integrated HHV-6 (iciHHV-6), who have the HHV-6 genome in every cell. It is known that telomeric repeats flanking the virus genome are essential for integration; however, the protein factors mediating integration remain enigmatic. We have previously shown that the putative viral integrase U94 is not essential for telomere integration; thus, we set out to assess the contribution of potential viral recombination proteins U41 and U70 towards integration. We could show that U70 enhances dsDNA break repair via a homology-directed mechanism using a reporter cell line. We then engineered cells to produce shRNAs targeting both U41 and U70 to inhibit their expression during infection. Using these cells in our HHV-6A in vitro integration assay, we could show that U41/U70 were dispensable for telomere integration. Furthermore, additional inhibition of the cellular recombinase Rad51 suggested that it was also not essential, indicating that other cellular and/or viral factors must mediate telomere integration.

## 1. Introduction

Human herpesvirus 6A and 6B (HHV-6A and 6B) are genetically closely related human herpesviruses. Primary infection with HHV-6B occurs in the first years of life and causes the febrile illness roseola infantum, which is occasionally accompanied by complications such as seizures and encephalitis [1,2]. The disease burden of HHV-6A remains poorly understood and humans appear to acquire this infection later in life. Interestingly, a recent study has highlighted a possible association between HHV-6A infection and Alzheimer’s disease [3], although more work is required to establish the nature of this association. After primary infection, HHV-6A/B establish a quiescent stage of infection termed latency. Both viruses have been shown to integrate their genome into the telomeres of latently infected cells [4,5,6]. Intriguingly, HHV-6 is also able to integrate into germ cells and can be transmitted vertically, resulting in ~1% of the world population harboring the virus in every nucleated cell [4,7,8,9,10,11]. Reactivation of HHV-6 from both the inherited and exogenously acquired forms has been associated with various severe diseases such as heart disease, graft versus host disease (GVHD), neuronal disorders and unexplained infertility [12,13,14,15,16,17,18,19].

Telomeric repeats (TMRs) present in the direct repeat regions (DRs) at the end of the virus genome are essential for efficient integration, suggesting that integration may occur by homologous recombination (HR) between the host telomeres and viral TMRs [20]. Surprisingly, we recently demonstrated that a mutant HHV-6A lacking the putative viral integrase (U94) efficiently integrated into host telomeres [21], suggesting that other viral or cellular factors facilitate the integration process.

Herpes simplex virus 1 (HSV-1) encodes two viral proteins, a single strand DNA-binding protein (ICP8; UL29) and a 5′-3′exonuclease [UL12], which are involved in recombination of the HSV-1 genome during replication [22,23,24,25]. These genes are highly conserved within the Herpesviridae family and the HHV-6A/B genomes contain putative orthologues of these two genes (termed *U41* and *U70*, respectively). As these viral proteins can mediate HSV-1 genome recombination, we hypothesized that the HHV-6A orthologues could facilitate virus integration. We were able to show that, similar to HSV-1 UL12, U70 enhances the single strand annealing (SSA) DNA repair pathway. Surprisingly, we could demonstrate that U70 and U41 were dispensable for HHV-6A telomere integration using cells expressing potent shRNAs. Moreover, as we utilized our U94-deletion virus, the lack of an effect on integration was not caused by functional redundancy between U94 and U41/U70. Strikingly, an additional block of the cellular recombinase Rad51 also did not inhibit telomere integration. Taken together, our data demonstrate that the HHV-6A integration mechanism is complex and indicate that other cellular DNA repair pathways and/or other viral proteins must facilitate this process.

## 2. Materials and Methods

### 2.1. Cell Culture and Viruses

JJhan cells were maintained in RPMI (PAN Biotech; Aidenbach, Germany) and supplemented with 10% fetal bovine serum (FBS; PAN Biotech) and penicillin/streptomycin. 293T cells were maintained in DMEM (PAN Biotech) supplemented with 10% FBS, penicillin, streptomycin and 5 µg/mL plasmocin (InvivoGen; Toulouse, France). HHV-6A U94-deletion GFP reporter virus (HHV-6A ∆U94) was described previously [21], and was propagated on JJhan cells.

### 2.2. Plasmid Construction and Lentivirus Production

To generate a lentiviral vector that delivers two shRNAs, we engineered pLKO5.sgRNA.EFS.PAC (Addgene; #57825). A synthetic construct containing two shRNA cloning sites and the 7SK promoter were obtained and inserted into the vector between the NdeI and EcoRI restriction sites. This yielded a vector containing a hU6-promoter-shRNA cloning site and an adjacent, although in the opposite orientation, 7SK promoter-shRNA cloning site. The shRNAs were generated by annealed oligonucleotide cloning and the sequences were inserted between the BsmBI sites (shRNA 1) and BfuAI sites (shRNA 2). The sequences are shown in Table 1.

Target sites were selected using the siRNA Wizard software (https://www.invivogen.com/sirnawizard/index.php) using the human off-target database to prevent off-targeting effects.

To change the antibiotic resistance gene in the pLKO5.shU41.PAC vector, the hygromycin resistance gene was amplified (Table 1) and inserted between the BamHI and MluI sites flanking the puromycin resistance gene.

The expression construct for HHV-6A U70 with a C-terminal HA-tag was produced by PCR amplification of the U70 coding sequence from the HHV-6A bacterial artificial chromosome (BAC) and was inserted into the pCMV-mGFP Cterm S11 Neo Kan vector (Sandia BioTech; Albuquerque, NM, USA) between the XhoI and BamHI sites (pU70-HA-S11). To insert a stop codon before the sequence coding for the C-terminal HA-tag, Phusion DNA polymerase site-directed mutagenesis (NEB) was performed according to the manufacturer’s instructions with the primers listed in Table 1. U41-HA was created by PCR amplification of the U41 coding region from the HHV-6A BAC and was inserted into psiCHECK-2 (Promega; Madison, WI, USA) between the NheI and XhoI sites (psiU41-HA), replacing the Renilla luciferase gene. All primer sequences are shown in Table 1.

Lentiviruses packaging the shRNA vectors were constructed by transfecting 293T cells with pCMVDR8.91, pCMV-VSV-G and either pLKO5.2XshU41.HPH or pLKO5.2XshU70.PAC. Lentiviruses were harvested 36 h post-transfection and used for transduction as described previously [26]. After the transduction of 293T cells with the lentiviruses expressing two shRNAs against U41 and U70 each, cells were selected with 1 µg/mL puromycin for 5 days, followed by the selection of cells with hygromycin 400 µg/mL for 7 days. From these polyclonal cells, single cell clones were produced by limiting dilution in 96 well plates.

### 2.3. 293T Single Strand Annealing (SSA) DNA Repair Reporter

To generate an SSA reporter cell line, we transfected 293T cells with the previously described hprtSAGFP reporter construct (Addgene; #41594 [27]), selected with 1 µg/mL puromycin for seven days and single cell clones were generated. The cells were maintained in 0.5 µg/mL puromycin, post-selection. These cells harbor a construct containing a disrupted GFP coding region that is separated by an I-SceI restriction site and flanked by homologous sequences that can only be repaired by SSA DNA-damage repair. Upon successful SSA repair, the GFP coding sequence is restored and GFP is expressed. To assess SSA enhancement by viral proteins, cells were PEI-transfected in duplicate with pCBA-SceI (Addgene; #26477), pE2-Crimson (Clonetech; Mountain View CA, USA) and the expression vectors: pU70-HA-S11, psiU41-HA, pU70ΔHA-tag, pSAKUL12, pSAKUL12-D340E or pcDNA3.1 (mock control; Invitrogen; Carlsbad CA, USA) at a molar ratio of 2:1:1. Three days post-transfection, the percentage of GFP (successful repair) and Crimson (transfected) positive cells were enumerated by flow cytometry. Repair was defined as $\frac{\left(Crimson+\&\;GFP+\right)}{\left(Crimson+\right)+\left(Crimson+\&GFP+\right)}$. Mock transfected cells were set to 1 and the other samples were expressed according to the mock for each independent experiment.

### 2.4. SDS-PAGE and Immunoblotting

Samples for protein analysis were lysed on ice using radioimmunoprecipitation (RIPA) buffers and benzonase. The samples were boiled, loaded, and separated by SDS-polyacrylamide gel electrophoresis. Proteins were transferred onto nitrocellulose membrane (Merck Millipore; Burlington, MA, USA) and blocked with 3% milk in PBS tween. These proteins were indirectly immunoblotted with anti-HA (1:1000; Cell Signaling; Danvers, MA, USA) and anti-β-actin (1:1000; Cell Signaling) followed by anti-mouse-HRP (1:10,000; Sigma–Aldrich; St. Louis, MO, USA) and anti-rabbit-HRP (1:5000; Cell Signaling) respectively. HRP was detected using Amersham ECL Prime (GE Healthcare; Chicago, IL, USA) using the Chemi-Smart 5100 detection system (PeqLab; Erlangen, Germany). The band intensity was quantified using BIO-1D v12 software (Vilber; Collegein, France) using the thresholding algorithm.

Samples for protein analysis were lysed on ice using radioimmunoprecipitation (RIPA) buffers and benzonase. The samples were boiled, loaded, and separated by SDS-polyacrylamide gel electrophoresis. Proteins were transferred onto nitrocellulose membrane (Merck Millipore; Burlington, MA, USA) and blocked with 3% milk in PBS tween. These proteins were indirectly immunoblotted with anti-HA (1:1000; Cell Signaling; Danvers, MA, USA) and anti-β-actin (1:1000; Cell Signaling) followed by anti-mouse-HRP (1:10,000; Sigma–Aldrich; St. Louis, MO, USA) and anti-rabbit-HRP (1:5000; Cell Signaling) respectively. HRP was detected using Amersham ECL Prime (GE Healthcare; Chicago, IL, USA) using the Chemi-Smart 5100 detection system (PeqLab; Erlangen, Germany). The band intensity was quantified using BIO-1D v12 software (Vilber; Collegein, France) using the thresholding algorithm.

### 2.5. In Vitro HHV-6 Integration Assay

The assay was performed as previously described [20,21]. Briefly, 293T or knockdown cells were co-cultured with HHV-6A infected JJhans for 4 h. JJhans were then washed away and the following day the GFP-positive cells sorted on a FACS AriaIII (BD Biosciences; Franklin Lakes, NJ, USA). Immediately post-sort, a sample was taken for DNA extraction and the remaining cells returned to the culture. The cells were passaged and 14 days post-sorting, a sample was taken for DNA isolation and another was prepared for fluorescent in situ hybridization as previously described [20,28,29].

### 2.6. RT-qPCR Analysis of U70 and U41 Levels during Infection

The cells were infected with equal doses of HHV-6A ∆U94 and the number of infected cells quantified by flow cytometry 24 h later. The following day, total RNA was extracted from the infected cells using the RNeasy kit (Qiagen; Venlo, Netherlands) and remaining DNA removed by DNase treatment (Promega). RNA was converted to cDNA using the Multiscribe reverse transcriptase (Applied Biosystems; Foster City, CA, USA) according to the manufacturer’s instructions. The qPCR was performed using the SensiFast master mix (Bioline; Memphis, TN, USA) on 5 µL of cDNA using the primers and TaqMan probes listed in Table 1. Relative levels of U70 and U41 RNA were normalized to the level of infection measured by flow cytometry.

### 2.7. qPCR Analysis

DNA was isolated from samples using the RTP DNA/RNA Virus Mini Kit (Stratec; Edmonton, AB, Canada) following the manufacturer’s guidelines. The qPCR was performed with FIREpol master mix (Solis BioDyne; Tartu, Estonia) on 1 µL of isolated DNA using the primers and TaqMan probes in Table 1. qPCR was performed on an ABI 7500 qPCR machine and analyzed using ABI StepOne software (Applied Biosystems; Foster City, CA, USA). 

### 2.8. HHV-6A Genome Fluorescent In Situ Hybridization (FISH)

The detection of the HHV-6A genome by fluorescent in situ hybridization (FISH) was performed as previously described [20,28,29]. However, to enhance the detection of the HHV-6A genome, the following modifications were applied. After washing off unbound anti-DIG FITC Fab fragments (Roche; Basel, Switzerland), cells were stained with a secondary mouse anti-FITC Alexa Fluor 488 antibody (1:200; Merck Millipore) for 20 min followed by the standard washing in detergent buffer (2× SSC 0.03% tween20). A tertiary anti-mouse Alexa Fluor 488 antibody (1:1000; ThermoFisher; Waltham, MA, USA) was then added for 20 min followed by washing. Images were acquired with a 100× objective on a Zeiss M1 microscope through Zeiss filters for DAPI and FITC using a metal halide lamp. The images were then analyzed using ImageJ (https://imagej.nih.gov/ij/).

## 3. Results

### 3.1. HHV-6A U41 and U70 in Single Strand Annealing (SSA) DNA Repair

**Figure 1:**
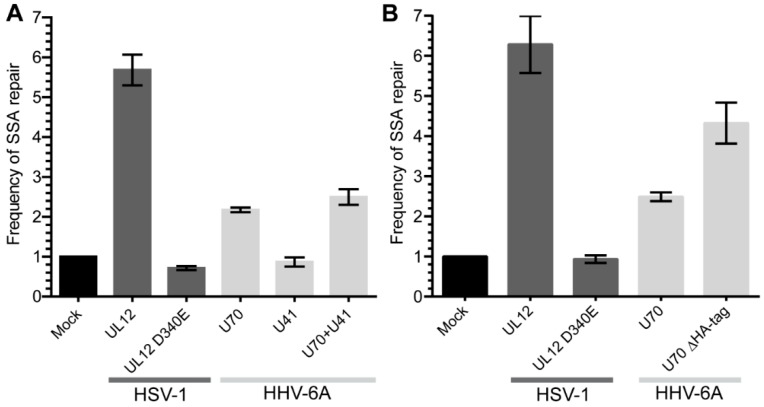
The role of HHV-6A U70 and U41 in double strand DNA break repair by single strand annealing (SSA). (**A**) An SSA reporter was integrated into 293T cells and the cells were transfected with the indicated expression plasmids. The frequency of repair was calculated and normalized to the mock transfected cells (control plasmid). Displayed are the mean frequency of repair for each transfected construct (*n* = 3 ± SEM). (**B**) The C-terminal HA-tag on U70 was removed and the same assay as in (**A**) was performed (*n* = 3 ± SEM).

HSV-1 UL12 has been shown to aid the repair of double strand DNA breaks (DSB) via a single strand annealing mechanism using a DNA damage reporter assay [23]. To test whether HHV-6A U70, the orthologue of HSV-1 UL12, can also facilitate DSB repairs through SSA, we utilized a well-established SSA reporter system in 293T cells [27]. The expression of HSV-1 UL12 significantly increased the SSA DNA repair that was abolished, by mutating the active site of the exonuclease (Figure 1), as previously reported [23]. Interestingly, HHV-6A U70 also enhanced DSB repair through the SSA pathway (Figure 1A). Furthermore, the removal of the C-terminal HA-tag on U70 increased its ability to aid SSA repair, suggesting that the tagging of U70 interferes with its activity (Figure 1B). The expression of HHV-6A U41 neither enhanced SSA repair nor augmented the effect of U70 (Figure 1A). Taken together, these data suggest that HHV-6A U70 can aid in the repair of homologous DNA sequences via the SSA pathway.

### 3.2. Generation of a U41 and U70 Knockdown Cell Line

**Figure 2:**
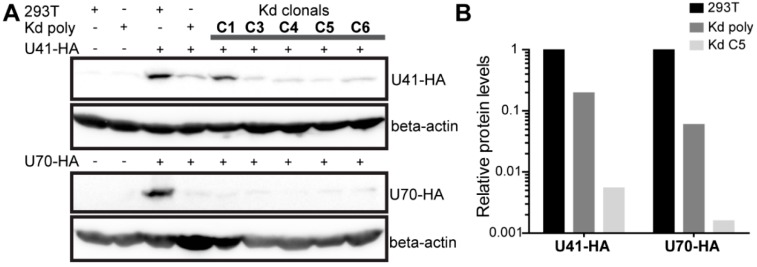
Validation of 293T HHV-6A U41/U70 knockdown cells. (**A**) 293T, Kd poly or single cell clones were individually transfected with U41-HA (upper panels) or U70-HA (lower panels) expression plasmids. One day post-transfection, cells were lysed and the proteins separated by SDS-PAGE. Immunoblotting was then performed using anti-HA or anti-beta-actin (beta-actin; loading control) antibodies. (**B**) Optical densities for HA bands were extracted using BIO-1D software and normalized for protein loading (β-actin). Displayed in the histogram is the viral protein expression in the poly clonal cells (Kd poly) and the best clonal cell (Kd C5) relative to expression in 293T control cells.

To determine if U70 and/or U41 are involved in HHV-6 integration, we set out to assess the integration efficiency upon the knockdown of both proteins. As UL12 and UL29 play a crucial role in HSV-1 replication [30,31,32], we generated target cells for our integration assay that express four shRNAs, two each against U41 and U70. Cells were selected after lentiviral delivery of the shRNAs against U41/U70 and the knockdown confirmed by Western blotting in the cells transfected with HA-tagged U41 or U70 expression plasmids. Polyclonal U41/U70 knockdown cells (Kd poly) displayed a robust knockdown of U41-HA and U70-HA compared to the parental 293T cells (Figure 2A), despite the high-level plasmid-driven overexpression of these viral genes. To achieve an almost complete knockdown, clones were isolated and tested for their knockdown efficiency as stated above (Figure 2A). Clone 5 (Kd C5) had the most efficient knockdown of both U41-HA and U70-HA (0.54% and 0.16% levels seen in 293T control, respectively) and was also included in our integration assay (Figure 2B).

In order to ensure that this U41/U70 knockdown was effective upon virus infection we assessed the levels of U41 and U70 RNA in knockdown cells infected with HHV-6A ∆U94. Both U41 and U70 RNA levels were reduced in both Kd cell lines (Figure S1). Taken together, these results suggest that the knockdown cells inhibit the expression of U41 and U70, even in the context of infection.

### 3.3. Effect of U41/U70 Knockdown on HHV-6 Integration

To assess the integration efficiency upon the knockdown of U41/U70, we used our HHV-6A integration assay as previously described [20,21]. We have previously shown that HHV-6A can efficiently integrate in the absence of the putative virus integrase (U94) [21]. However, since U94 was still able to compensate for the loss of U41/U70, we used our U94-deletion HHV-6A virus (HHV-6A ∆U94) to assess the integration in the U41/U70 knockdown cells.

**Figure 3:**
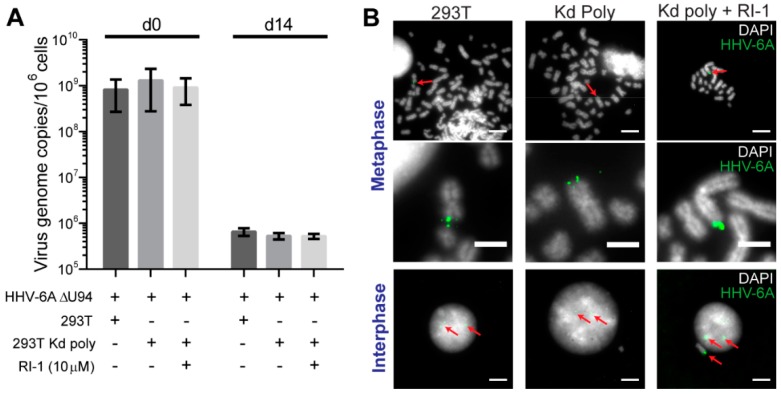
The effect of HHV-6A U41/U70 knockdown on HHV-6A integration. 293T or Kd polyclonal (Kd poly) cells were infected with the HHV-6A ∆U94 virus and the GFP-positive cells sorted. Samples were taken immediately after sorting and 14 days later. (**A**) Mean virus DNA copies per million cells was quantified by qPCR using primers against viral U86 and cellular B2M. This number is displayed in the histogram (*n* = 4 ± SEM). RI-1 is a specific inhibitor of the Rad51 cellular recombinase. (**B**) The 14-day samples were analyzed by FISH to detect the HHV-6A genome (green). Representative images are displayed for metaphase and interphase cells (DAPI staining shown in grey). Red arrows indicate the location of HHV-6A signals. Scale bars for the top and bottom images are 10 µm and 4 µm for the images in the middle.

293T or Kd poly cells were infected, cultured over-night and sorted to obtain a pure GFP-positive-infected population. Samples were taken post-sort and 14 days later to quantify the virus genome maintenance within the culture. After sorting, virus DNA levels were comparable for all samples (Figure 3A). Strikingly, the virus genome maintenance over time was not altered upon the knockdown of U41/U70, suggesting that the expression of both proteins was not essential for the HHV-6A maintenance in cell culture. To determine if the cellular recombinase Rad51 can complement for the loss of U41, U70 and U94, we also inhibited Rad51 using a small molecule inhibitor. However, HHV-6A was still efficiently maintained in the absence of all three viral proteins and activity of the cellular recombinase (Figure 3A). To confirm the integration of the HHV-6A genome at the ends of host chromosomes, we performed fluorescent in situ hybridization (FISH). HHV-6A was readily detected at chromosome ends in the absence of all three viral proteins and inhibition of the cellular recombinase Rad51 (Figure 3B), indicating that all four proteins were dispensable for HHV-6A integration. 

**Figure 4:**
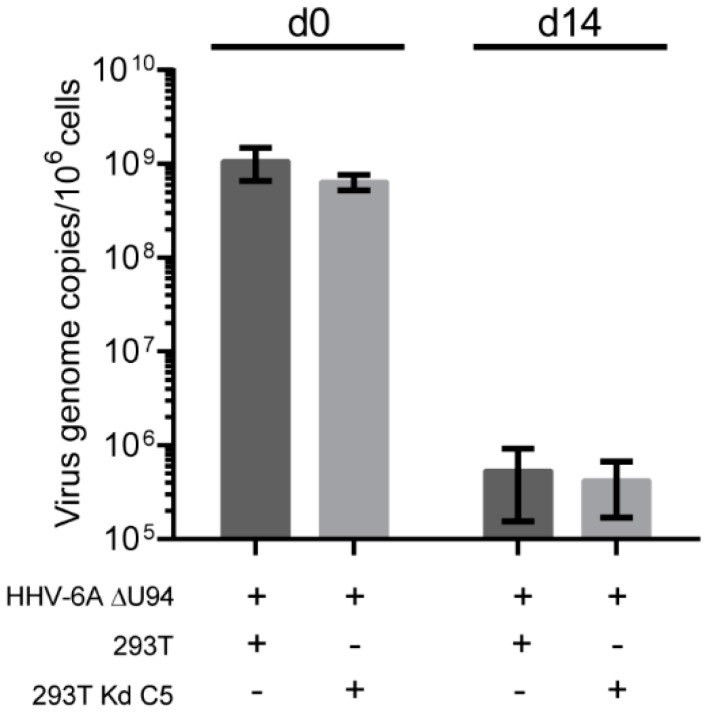
The effect of HHV-6A U41/U70 knockdown on telomere integration in a clonal knockdown cell line. The integration assay was performed as described in Figure 3A but using the U41/U70 single cell clone (Kd C5). Mean virus DNA copies per million cells was quantified by qPCR against viral U86 and cellular B2M. This number is shown in the histogram (*n* = 3 ± SEM).

To confirm that the observed phenotype in the Kd poly cells was not due to some cells within the population with a partial knockdown phenotype (Figure 2A), we performed the same integration assay with Kd C5. As observed for the Kd poly cells, the knockdown of U41/U70 did not inhibit HHV-6A genome maintenance even in the absence of U94 (Figure 4). Taken together, our data demonstrate that the viral proteins U41, U70 and U94, as well as the cellular recombinase Rad51 are dispensable for HHV-6A integration. 

## 4. Discussion

HHV-6A and -6B have been shown to integrate their genome into the host chromosomes of latently infected cells. We have recently shown that the viral telomere sequences are vital for this integration process; however, the protein counterparts remain unknown. It had been proposed that the putative integrase U94 facilitates integration based on the fact that it interacts with telomere DNA, possesses the enzymatic activities necessary to mediate recombination and could facilitate the replication of adeno-associated virus 2 (AAV-2) lacking its Rep68 integrase [33,34]. However, we recently demonstrated that HHV-6A lacking U94 efficiently integrated into host chromosomes in vitro [21]. Multiple possibilities exist as to how the virus could integrate in the absence of U94: (i) U94 is not the viral integrase (ii) redundant pathways exist for telomere integration or (iii) the integration pathway in vivo differs from the one utilized in vitro. In this study we set out to address (i) and (ii) by targeting the HHV-6A proteins U41/U70 that could potentially facilitate integration in the absence of U94.

*U41* and *U70* are the orthologues of *UL29* and *UL12* from HSV-1 that encode a single strand DNA binding protein (UL29) and a 5′-3′ exonuclease (UL12), respectively. These genes are highly conserved for the Herpesviridae and have been shown to facilitate the recombination of the HSV-1 genome via the SSA DNA repair pathway [23,24,25]. Since all available data suggest that HHV-6 integrates by recombination between the viral and host telomeres, U41/U70 could potentially be involved in this process by augmenting SSA.

We could demonstrate that U70 enhances the SSA DNA repair pathway using a well-established reporter system (Figure 1), although the magnitude of the effect was smaller than that seen with HSV-1 UL12 [23]. Interestingly, the removal of the C-terminal HA-tag on U70 showed that the tag impaired U70 function. However, the activity of U70 in SSA remained weaker than its counterpart from HSV-1 (Figure 1B).

Intriguingly, we were able to demonstrate that HHV-6A efficiently integrated upon the knockdown of U41/U70 (Figure 3 and Figure 4). This was shown using HHV-6A lacking U94 to ensure that it did not substitute for the loss of U41/U70 expression. The additional inhibition of homologous recombination (HR) using RI-1 did not significantly reduce the ability of HHV-6A to integrate into host chromosomes (Figure 3), indicating that it does not compensate for the loss of these three viral factors. Taken together, our data shows that HHV-6A U70 can aid in SSA DNA repair but U41/U70 are dispensable for HHV-6A integration and do not compensate for the loss of U94 or cellular HR DNA repair.

Although we demonstrated that the most likely viral candidates and HR are dispensable for HHV-6A integration, the factors that facilitate this process still remain unknown. Cells possess more than one pathway that can mediate a recombination and repair breaks in the genome. Some of these pathways are specialized in the repair of homologous DNA sequences and we will investigate if these pathways are involved in HHV-6 integration in future studies. The delineation of the factors involved in HHV-6 integration will uncover the integration mechanism and unveil the process that permitted this virus to enter the human germ line.

## Figures and Tables

**Table 1:** Oligonucleotide sequences used in this study.

Cloning Step/qPCR		Sequence (5′ → 3′)
shRNA U70#1	For	CACCGGAGTGGATGGATCGGAAGACATCAAGAGTGTCTTCCGATCCATCCACTCTTTTTT
	Rev	CAGCAAAAAAGAGTGGATGGATCGGAAGACACTCTTGATGTCTTCCGATCCATCCACTCC
shRNA U70#2	For	ATCAAAAAAGACGGCGACTAAGTTGTATGACTCTTGATCATACAACTTAGTCGCCGTCGAGG
	Rev	GGTACCTCGACGGCGACTAAGTTGTATGATCAAGAGTCATACAACTTAGTCGCCGTCTTTTT
shRNA U41#1	For	CACCGGTTTCTGCTCCCGTTTCTACTTCAAGAGAGTAGAAACGGGAGCAGAAACTTTTTT
	Rev	CAGCAAAAAAGTTTCTGCTCCCGTTTCTACTCTCTTGAAGTAGAAACGGGAGCAGAAAC
shRNA U41#2	For	ATCAAAAAAAGATTTCTCGACCACGGTTAAACTCTTGATTTAACCGTGGTCGAGAAATCGAGG
	Rev	GGTACCTCGATTTCTCGACCACGGTTAAATCAAGAGTTTAACCGTGGTCGAGAAATCTTTTTT
Puromycin to hygromycin resistance gene	For	GCATGGATCCGCCACCATGAAGAAACCTG
	Rev	CTGCACGCGTTCATTCCTTGGCTCTGGG
U70-HA amplification for cloning	For	CTACACTCGAGGCCACCATGGATCTTGATCAAATATCTGAAACAC
	Rev	CTCAGGGATCCACCGCATAATCCGGCACATCATACGGATAACTACCACCAGGTGTTTTCGGTTTTCTTACACATG
U41-HA amplification for cloning	For	TTAGCTAGCCACCATGGCTGATGAAAACG
	Rev	TGTACTCGAGTTACGCATAATCCGGCACATC
Stop insertion SDM for U70	For	GAAAACACCTTAAGGTGGTAGTTATC
	Rev	GGTTTTCTTACACATGCCGC
ß2M qPCR	For	CCAGCAGAGAATGGAAAGTCAA
	Rev	TCTCCATTCTTCAGTAAGTCAACTTCA
	Probe	*FAM*-ATGTGTCTGGGTTTCATCCATCCGACA-*TAMRA*
U86 qPCR	For	TGTACATGGGCTGTAGGAGTTGA
	Rev	ACATCCTCTGCTTCCAATCTACAATC
	Probe	*FAM*-TTCCGAAGCAAAGCGCACCTGG-*TAMRA*
U70 qPCR	For	GGGCCGTAAACTTATTGAGG
	Rev	CAGCTTGCACAATTCACTCA
	Probe	*FAM*-TCCATCCACTCTTGTGCCGCA-*TAMRA*
U41 qPCR	For	CACGATTGACAACATTTCCC
	Rev	GGGTAATGCGCATACTGAGA
	Probe	*FAM*-TCGCCGAACAATTTACCAGATGATTG-*TAMRA*

## Supplementary Materials

The following are available online at http://www.mdpi.com/1999-4915/10/11/656/s1,Figure S1: U41 & U70 mRNA levels in knockdown cells.
