# Supplementary material for: Viral Proteins U41 and U70 of Human Herpesvirus 6A Are Dispensable for Telomere Integration
Source: Viruses. 2018 Nov 21;10(11):656. doi: 10.3390/v10110656 (PMC6267051; doi:10.3390/v10110656)
Supplement: Supplementary file 1 [file viruses-10-00656-s001.pdf]

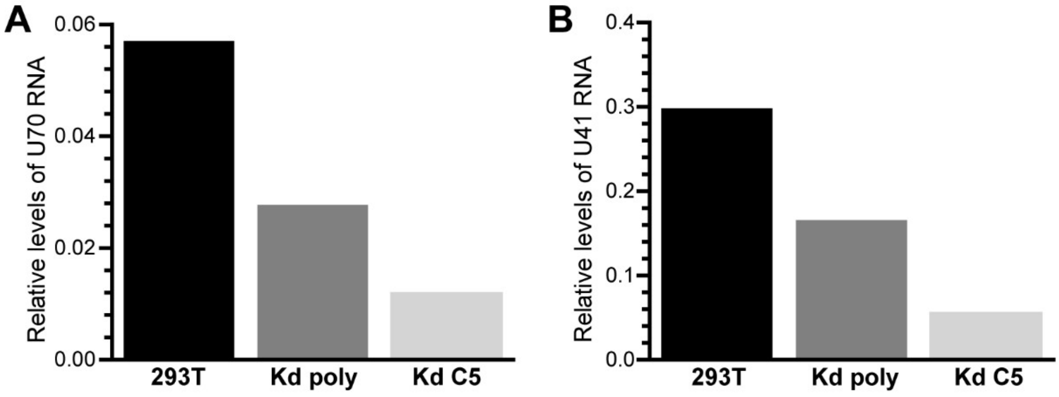

Figure S1. U41 & U70 mRNA levels in knockdown cells. 293T, Kd poly or Kd C5 were infected with HHV-6A dU94 and 2 days later RNA was isolated. RT-qPCR was performed using primers against U41, U70 and B2M. Levels of U41 and U70 RNA were normalized by infection rate and are displayed relative to levels of B2M RNA. Data is from one representative experiment ($n = 2$).
